# EcoSun Pass: A tool to evaluate the ecofriendliness of UV filters used in sunscreen products

**DOI:** 10.1111/ics.12681

**Published:** 2021-01-20

**Authors:** S. Pawlowski, B. Herzog, M. Sohn, M. Petersen‐Thiery, S. Acker

**Affiliations:** ^1^ BASF SE GBP/RA Z 570 Ludwigshafen Germany; ^2^ BASF Grenzach GmbH EMR/CU Global Development UV Protection/Scientific Liaisons Grenzach‐Wyhlen Germany; ^3^ BASF Personal Care and Nutrition GmbH E‐EMC/QR Product Stewardship & EHS Data Management Monheim Germany

**Keywords:** sun care, UV protection, melanogenesis, formulation skin care, ecotox, ecofriendly, emulsions

## Abstract

**Objective:**

Sunscreens play a major role in the EU sun protection strategy in order to prevent humans from UV light‐induced skin damage. In recent years, the demand for high‐quality sunscreen products including aspects of broad range and photostability of the UV protection, showing good spreadability onto human skin and excellent sensorial properties during and after application has increased. Environmental aspects are considered. Sunscreens are complex compositions, with UV filters being the key element in the formulations reaching up to about 30% in content in the final product. Some of these ingredients, however, may be regarded as hazardous for the aquatic environment. Nevertheless, the aquatic ecosystem represents only a single environmental compartment, which may be impacted by UV filters. Therefore, the EcoSun Pass (ESP) tool was developed in order to assess the overall environmental impact of UV filters in combination with its efficacy (Sun Protection Factor, SPF and UVA Protection Factor, UVA‐PF).

**Methods:**

For that purpose, at first 24 of the EU‐approved UV filters for sunscreen applications were evaluated for their environmental hazard profiles. Nine example UV filter compositions representing both SPF 30 and 50 were evaluated for ecofriendliness using the ESP tool.

**Results:**

The results revealed that two out of four SPF 30 compositions are considered as ecofriendly. Likewise, from the SPF 50 two out of five did meet the criteria for ecofriendliness. Furthermore, the results showed that most ecofriendly example formulations have also the lowest overall UV filter content in the product, based on the use of highly innovative and least hazardous UV filters.

**Conclusion:**

These results demonstrate that the tool is applicable to various formulations being present on the market and thus allows for a selection of most ecofriendly and efficient UV filters to be used in sunscreens.

## Résumé

**Objectif:**

les écrans solaires jouent un rôle majeur dans la stratégie de protection solaire de l’UE, afin de protéger les êtres humains contre les lésions cutanées causées par les rayons ultraviolets. Au cours des dernières années, la demande pour des écrans solaires de haute qualité a augmenté, notamment ceux ayant une bonne capacité d’étalement sur la peau humaine, d’excellentes propriétés sensorielles pendant et après l’application, une plage large et démontrant la photostabilité de la protection anti‐UV. Les aspects environnementaux sont pris en compte. Les écrans solaires ont des compositions complexes, les filtres UV étant l’élément clé des formulations, avec une présence jusqu’à près de 30 % dans le contenu du produit final. Toutefois, certains de ces ingrédients peuvent être considérés comme dangereux pour l’environnement aquatique. Néanmoins, l’écosystème aquatique ne représente qu’un seul compartiment environnemental pouvant être affecté par les filtres UV. Par conséquent, l’outil EcoSun Pass (ESP) a été développé afin d’évaluer l’impact environnemental global des filtres UV, ainsi que leur efficacité (facteur de protection solaire, FPS et facteur de protection UV‐A, UVAPF).

**Méthodes:**

dans ce but, les profils de risque environnemental de 24 des filtres UV approuvés dans l’UE, pour les applications d’écran solaire, ont d’abord été évalués. Le respect de l’environnement de neuf exemples de compositions de filtres UV, représentant les FPS 30 et 50, a été évalué à l’aide de l’outil ESP.

**Résultats:**

les résultats ont révélé que deux des quatre compositions de FPS 30 sont considérées comme écologiques. De même, deux écrans solaires sur cinq ayant un indice FPS 50 répondaient aux critères de respect de l’environnement. De plus, les résultats ont montré que la plupart des exemples de formulations écologiques contiennent également la plus faible teneur globale en filtres UV ; elles sont basées sur l’utilisation de filtres UV hautement innovants et les moins dangereux.

**Conclusion:**

ces résultats démontrent que l’outil est applicable à diverses formulations présentes sur le marché, et qu’il permet donc d’utiliser une sélection des filtres UV les plus écologiques et efficaces dans les écrans solaires.

## Introduction

Cosmetic products are widely used around the globe and the demand for high‐quality products is constantly increasing. This high quality includes both functional and performance aspects such as broad range absorption and photostability of the UV protection, showing good spreadability onto human skin and excellent sensorial properties during and after application.

Sunscreens as part of the entire cosmetic portfolio, play a major role in the European Union’s (EU) sun protection strategy to prevent humans from ultraviolet (UV) light‐induced skin ageing and skin cancer [1, 2]. Also, the World Health Organization (WHO) developed a teaching strategy including the use of sunscreens in order to protect especially children as the most vulnerable group from long‐term skin damage [3].

However, these types of personal care products are likely to enter the environment in a direct manner due to leisure activities such as swimming in lakes, rivers and coastal areas or even through sunbathing on green land [4, 5, 6, 7, 8]. Furthermore, indirect exposure to the terrestrial environment may occur, due to so‐called ‘sludge to soil’ applications [9, 10, 11, 12]. On the other hand, field workers who are daily exposed to sunlight need additional sun protection using highly efficient sunscreen products [13, 14]. A human safety assessment of cosmetics is required in the European Union. UV filters as key ingredients in sunscreen products furthermore need separate approval of the European Commission’s scientific board Scientific Committee on Consumer Safety (SCCS) prior to getting on the EU market. Only filters, which are considered as safe during application will be listed on the positive list for UV filters – ANNEX VI of the Cosmetics Regulation (EC) No. 1223/2009, so that they can be used in sunscreen formulations [15]. However, in recent years, the demand for environmentally friendly and sustainable pharmaceuticals, cosmetics and sunscreen products has increased [16].

In many countries, cosmetics are treated differently to other industrial chemicals. However, as many of the ingredients in a common cosmetic preparation have additional functions outside the cosmetic use, these substances have to obey also the chemicals inventory requirements [17, 18, 19, 20, 21]. Often, specifically UV filters for use in sunscreens are exempted from chemical registration as they have beneficial aspects to human health (i.e. Japan, Korea, Australia, United States, Canada). In these countries, UV filters have to undergo a specific registration process similar to the European Union, but sometimes even require the higher standards of pharmaceutical registration (United States, Canada, Australia). Also, the compositions of sunscreens may differ between countries due to different perceptions of the customers. Therefore, companies selling sunscreens in the global marketplace must be able to readily determine whether their products meet the requirements of a particular country.

Various techniques have been developed to assess the environmental impact that formulations may have, for example, a ‘grading system’ based on a supplier’s environmental practices, categorization ingredients to determine an environmental ‘footprint’ of a product, or a ‘grading system’ based solely on the toxicity of individual components and the environmental impact of a product. However, such techniques do not take into consideration all the available experimental physical–chemical, ecotoxicological and environmental fate data for a particular component when assessing the environmental hazard of a formulation. Other evaluation systems focus on the aquatic compartment or environmental classification and labelling (again, restricted top aquatic toxicity). For certain chemicals such as UV filters used in sunscreen products, a more holistic approach including all environmental relevant compartments is deemed to be necessary [22]. Within the EU, ecolabel criteria have been developed for rinse‐off products only, whereas sunscreens are considered as leave‐on products [23]. These criteria are furthermore limited to ecotoxicological and environmental fate to the aquatic compartment following the principals of classification and labelling. However, the environmental impact of UV filters used in sunscreen products has been increasingly examined due to potential damaging effects that certain filters may have on the environment [24, 25, 26, 27]. Thus, there remains a need for the evaluation of the environmental impact of individual sunscreen components such as UV filters to determine the overall ecological behaviour of a composition containing certain substances. Therefore, a science‐based tool, entitled EcoSun Pass (ESP) was developed which considers beneath the environmental impact of an UV filter, the concentration in the product and its efficiency in order to select for the components with the best ecological footprint.

## Material and methods

### Composition of sunscreens and rationale for this approach

Sunscreen formulations are complex mixtures typically containing five to six individual UV filters, in addition to emollients, emulsifiers, thickeners, preservatives, boosters, sensory enhancers and preservatives [28]. A combination of various UVA, UVB and broad‐spectrum filters is needed to cover the relevant UV light range from 290 to 400 nm as well as the very high sun protection factor (SPF) of ≥50. High sun protection is mandatory in sunlight intensive regions such as Mediterranean region, central America, Australia, Asia or Chile [28, 29, 30].

The fact that sunscreens are mixtures, allows to select for ingredients with the lowest environmental impact for the respective formulation.

### Substances evaluated

In order to evaluate the suitability of the ESP tool, approved and registered UV filters according to the EU cosmetics regulation are used, which currently comprises 28 active substances, 24 of which were assessed in the current evaluation (Table 1).

**Table 1:** Overview of EU‐approved UV filters used in cosmetic sunscreen products.

INCI name and Abbreviation	USAN	<E11>^1^	CAS N°
4‐Methyl benzylidene camphor (MBC)	Enzacamene	275	36861‐47‐9
Benzophenone‐3 (B‐3)	Oxybenzone	237	131‐57‐7
Benzophenone‐4 (B‐4)	Sulisobenzone	168	4065‐45‐6
Bis‐ethylhexyloxyphenol methoxyphenyl triazine (BEMT)	Bemotrizinol	527	187393‐00‐6
Butyl methoxy dibenzoyl methane (BMDBM)	Avobenzone	571	70356‐09‐1
Terephtalidene dicamphor sulphonic acid (TDSA)	Ecamsule	400	92761‐26‐7
Diethylamino hydroxybenzoyl hexyl benzoate (DHHB)	‐	351	302776‐68‐7
Diethylhexyl butamido triazone (DBT)	‐	451	154702‐15‐5
Disodium phenyl dibenzimidazole tetrasulfonate (DPDT)	Bidisulizole disodium	366	180898‐37‐7
Drometrizole trisiloxane (DTS)	‐	210	155633‐54.8
Ethylhexyl dimethyl PABA (ED‐PABA)	Padimate‐O	273	21245‐02‐3
Ethylhexyl salicylate (EHS)	Octisalate	53	118‐60‐5
Ethylhexyl triazone (EHT)	‐	420	88122‐99‐0
Ethylhexylmethoxy cinnamate (EHMC)	Octinoxate	271	83834‐59‐7
Homomenthyl salicylate (HMS)	Homosalate	46	118‐56‐9
Isoamylmethoxy cinnamate (IMC)	Amiloxate	325	71617‐10‐2
Methylene bis‐benzotriazolyl tetramethyl butylphenol (MBBT)	Bisoctrizole	361	103597‐45‐1
Octocrylene (OCR)	Octocrylene	142	6197‐30‐4
Phenyl benzimidazole sulphonic acid (PBSA)	Ensulizole	251	27503‐81‐7
Polysilicone 15 (BMP)	‐	59	207574‐74‐1
Titanium dioxide (TiO2)	Titanium dioxide	373	13463‐67‐7
Tris‐biphenyl triazine (TBPT)	‐	581	31274‐51‐8
Phenylene bis‐diphenyltriazine	‐	520	55514‐22‐2
Zinc oxide (ZnO)	Zinc oxide	98	1314‐13‐2

INCI, international nomenclature of cosmetic ingredients; USAN, US adopted name.

^1^ Averaged specific extinction of the active material <E11> in the spectral range between 290 and 400 nm.

Furthermore, for sunscreen typical emollients, thickeners and preservatives were evaluated. However, as the characteristics and the hazard profiles of these non‐UV active substances are very similar, only marginal differences between the individual components were identified. Typically, those substances are readily biodegradable, have a low acute aquatic toxicity, a low log octanol‐water partition coefficient (logPow) and a low adsorption potential and thus higher Tier tests on aquatic organisms (e.g. chronic toxicity, bioaccumulation) or even tests on sediment or soil‐dwelling organisms are not required according to chemical law (e.g. REACH). Because of the very similar substance properties of, that is emollients, only slight differences (if any) in acute aquatic toxicity will drive the EcoSun Pass evaluation, whereas sediment and soil toxicity are not of concern as these components will not end up in those compartments (i.e. sediment and soil). Furthermore, efficacy such as described for UV filters (see below for details) could not be applied to these co‐formulants.

A proper ecotoxicological ranking within the groups of emollients, thickeners and preservatives was not possible. Therefore, the evaluation of the ecotoxicological hazard ranking of the individual substances was focused to UV filters only.

### Efficacy of UV filters

In this work, the average specific extinction in the spectral range between 290 and 400 nm of a UV filter is employed as a measure of its efficacy. Specific extinction is given as the extinction one would observe with a 1 wt% solution or dispersion of the UV filter substance at an optical pathlength of 1 cm and is therefore designated as E11. For the average of the latter in the spectral range between 290 and 400 nm the symbol <E11> will be used. For achieving a certain protective effect, filters having a rather low efficacy will lead to higher amounts within a cosmetic formulation compared to UV filters having a higher efficacy. Within the EU region, currently 28 organic and inorganic UV filters are approved to be used in cosmetic sunscreen products. The names and efficacies of the 24 EU‐approved UV filters investigated are listed in Table 1 with efficacies ranging from 46 (homomenthyl salicylate, HMS) at the lower end to 581 (tris‐biphenyl triazine, TBPT) at the higher end.

### Data sources

For the evaluation of the hazard profile of the individual components, the following data sources were researched: (1) study reports (if available); (2) scientific‐based literature (if available); (3) information from governmental authorities (e.g. European Chemicals Agency –‘ECHA’ website) (if available); and (4) Quantitative Structure Activity relationship (QSAR) calculations (e.g. US EPA, Estimation Programs Interface Suite™ for Microsoft® Windows, v4.11. United Stated Environmental Protection Agency, Washington, DC, USA; [31]). Available study reports on substances were selected based on CAS number and study type. As a second step, scientific literature was screened by CAS numbers or substance names in combination with a selection of adequate key words. ECHA‐Website information was screened according to the identification parameters of the individual substance (i.e. CAS or EC number). QSAR estimations were carried out using simplified molecular‐input line‐entry system (SMILES) codes derived from the available chemical structures or the CAS number itself.

The quality of the data was furthermore assessed using the criteria as set within the Klimisch code in order to conclude on the validity of test results [32].

The data collection includes preferably experimentally derived physical–chemical, ecotoxicological and environmental fate data. Most of these data follow OECD or equivalent test guidelines and Good Laboratory Principles (GLP); in cases where no experimental data were available, additional data based on Quantitative Structural Activity Relationship (QSAR) were used instead. The use of QSAR data, however, is of limited value for the hazard profile derivation. It is more appropriate for a few selected endpoints such as logarithmic octanol‐water partitioning coefficient (log Pow) and bioaccumulation. The assessment of chronic aquatic toxicity or terrestrial toxicity by means of QSAR, was not considered adequate, given the high uncertainty in the calculated results [33].

### Evaluation of the environmental hazard score

In order to evaluate the environmental hazard score of the individual UV filters (both organic and inorganic), all available data were clustered according to the following environmental endpoints: biodegradation, bioaccumulation, acute aquatic toxicity, chronic aquatic toxicity, sediment toxicity and chronic terrestrial toxicity.

A ranking of the individual substance is based on its environmental fate and ecotoxicological profile. Criteria were defined for each environmental part and the available data within these chapters were ranked with a scoring number (Table 2). Components with the best environmental behaviour in a specific environmental segment were indicated with the lowest number (0.25), whereas substances having the most negative impact for the respective endpoint were rated with the highest number (i.e. 1.0).

**Table 2:** Environmental chapters, relevant criteria and related scores.

Environmental chapter	Pass level^1^	Factor
Biodegradation	Readily biodegradable/not persistent	0.25
	Biodegradable/not persistent	0.5
	Partly biodegradable/potential persistent	0.75
	Poorly biodegradable/persistent	1
Bioaccumulation	Bioaccumulation factor (BCF) <500	0.25
	Bioaccumulation factor (BCF) ≥500 ‐ <2000	0.5
	Bioaccumuation factor (BCF) ≥2000 ‐ <5000	0.75
	Bioaccumulation factor (BCF) >5000	1
Acute aquatic toxicity	EC_50_ > 100 mg/L or >water solubility	0.25
	EC_50_ ≤ 100 ‐ ≥10 mg/L	0.5
	EC_50_ ≤ 10 ‐ ≥1 mg/L	0.75
	EC_50_ < 1 mg/L	1
	EC_50_ 10 times lower as previously	+0.25
Chronic aquatic toxicity	NOEC/EC_10_ ≥ 10 mg/L	0.25
	NOEC/EC_10_ ≤ 10 ‐ ≥1 mg/L	0.5
	NOEC/EC_10_ ≤ 1 ‐ ≥0.1 mg/L	0.75
	NOEC/EC_10_ < 0.1 ‐ ≥0.01 mg/L	1
	NOEC/EC_10_ 10 times lower as previously	+0.25
Chronic terrestrial toxicity	NOEC/EC_10_ ≥ 1000 mg/kg dw	0.25
	NOEC/EC_10_ ≤ 1000 ‐ ≥100 mg/kg dw	0.5
	NOEC/EC_10_ ≤ 100 ‐ ≥10 mg/kg dw	0.75
	NOEC/EC_10_ < 10 ‐ ≥1 mg/kg dw	1
	NOEC/EC_10_ 10 times lower as previously	+0.25
Sediment toxicity	NOEC/EC_10_ ≥ 1000 mg/kg dw	0.25
	NOEC/EC_10_ ≤ 1000 ‐ ≥100 mg/kg dw	0.5
	NOEC/EC_10_ ≤ 100 ‐ ≥10 mg/kg dw	0.75
	NOEC/EC_10_ < 10 ‐ ≥1 mg/kg dw	1
	NOEC/EC_10_ 10 times lower as previously	+0.25

EC_10_/EC_50_ – effect level at 10 and 50%, respectively; NOEC – No Observed Effect Concentration; dw – dry weight.

^1^ Readily biodegradable: readily biodegradable according to OECD criteria.

To provide a comparable scale for the assessment of the different environmental compartments, each chapter starts with 0.25 as the lowest value, which is increased with higher specific toxicity in steps of 0.25. For the two environmental fate related chapters (i.e. biodegradation and bioaccumulation), the highest achievable (‘worst case’) value is 1.0, whereas for the ecotoxicology‐related endpoints, an upper (highest) threshold value is not sensible. Nevertheless, an upper value of 1.0 may be sufficient to assess the ecotoxicological effects of most components. For ecotoxicological chapters, the lowest score was applied to all substances showing no effects up to the highest concentration tested (as recommended by the corresponding OECD test guideline) or up to the limit of solubility under test conditions.

In cases where no adequate (higher Tier) data or QSAR based information are available, basic core data of the component like physical–chemical and environmental fate properties were used to fill the data gap in order to have an equal data basis for evaluation of all filters. In fact, a substance with a log Pow of ≥4.5 was rated bioaccumulative/very bioaccumulative if adequate experimentally derived or valid estimated bioaccumulation data were not available. In contrast, a substance with a log Pow <4.5 was considered as not bioaccumulative and not very bioaccumulative. In addition, a substance indicating poor biodegradability in the OECD 301 and OECD 302 [34, 35] tests and having the potential to adsorb to organic matter (as indicated by a high Log Pow or log Koc ([organic carbon to water partition coefficient]) is likely disseminating to both to sediment and soil. Therefore, additional data on sediment and terrestrial organisms are needed to address the concern adequately. In the absence of such data, a worst‐case toxicity assessment was applied to these compartments. In case the screening criteria for adsorption indicate a low adsorption potential of the substance (as indicated by a low Log Pow and or a low Log Koc‐value), and/or the substance is known to be readily biodegradable, no indirect transfer to the soil and sediment was assumed and thus, no additional data for the soil and sediment compartment are considered to be relevant. Then, a best‐case conclusion for compartments with missing adequate data was applied.

Due to the expected half‐life of UV filters in both soil and sediments in the range of at least a few weeks (according to the Technical guidance Document on Risk Assessment, European Commission, 2003, part II), chronic toxicity tests instead of acute toxicity tests are more appropriate. Available acute toxicity tests on both soil and sediment organisms are only rated as indicative but not as definitive for the assessment. For inorganic UV filters, the biodegradation criteria are not applicable as they do not degrade in the environment and thus their environmental concentrations may increase over time leading to long‐lasting effects. However, even if are considered as ‘naturally occurring’ in some jurisdictions, it is well known that some of the inorganic UV filters (i.e. zinc oxide, a relevant trace element for many living organics) have also acute and chronic effects on a great number of species found in various environmental compartments (see ECHA REACH dossier on zinc oxide for more details). Thus, increasing concentrations of inorganic UV filters in the environment over time may lead to ecotoxicological relevant (i.e. toxic) concentrations in the future. An example may be in the higher copper content in the soil of certain regions in Germany as a result of the application of the copper‐based ecofriendly plant protection products [36, 37]. In cases were basic core data were unavailable for one or more environmental chapters of a substance, either a best or a worst‐case assumption was made, which yielded either in the lowest or the highest reasonable scoring (i.e. 1) for the environmental segment for which data are missing. As will be described in more detail below, a relatively higher score indicates that a component has relatively poorer environmental characteristics, a lower scoring indicates a better environmental profile.

### Determination of the overall hazard score

The environmental profile of a substance is used to determine the overall environmental hazard score for the substance. As discussed above, there are six environmental segments each having a score ranging from 0.25 to typically 1.0 or 1.25. To determine the overall environmental hazard score of an individual substance, the scores in each of the environmental segments are added. The overall environmental scoring range is hence between 1.5 (ecofriendly) and 7.0 (hazardous).

In order to give an indication of the uncertainty of the overall environmental hazard score based on experimentally derived or QSAR based environmental data, a range was specified, designated as ‘real’, ‘best’ and ‘worst case’. ‘Real’ refers to the current evaluation status of the substance based on all available and reliable data and conclusion drawn thereof. The best and worst cases refer to the possible scenarios which are obtained, when the lacking ecotoxicological data are assumed to be most or least advantageous for the hazard score, respectively. When new data become available, this will lead to a narrowing of the spread between the best and worst cases, and at the same time contribute to the ‘real’ case resulting in lower uncertainty. Nevertheless, despite that there is some variability in the amount of data available for the individual UV filters, the existing information allows for conclusions on all six environmental segments, even if they may be considered as preliminary due to ongoing studies or even due to missing experimental data at the time the evaluation was made. In case of missing experimental or QSAR based information, expert judgement was used to draw a conclusion on any missing chapter.

### Cut‐off criteria

Substances meeting the criteria for being an endocrine disruptor (ED; according to the WHO definition), being acute (EC/LC_50_ < 0.1 mg/L) or chronically toxic towards aquatic organisms (NOEC/EC_10_ < 0.01 mg/L; according to the ECHA criteria for toxicity – T), or substances which are confirmed to meet the ECHA criteria for being persistent, bioaccumulative and toxic (PBT) or very persistent and very bioaccumulative (vPvB), are rated as not ecofriendly at all and thus not are considered as appropriate for further ecofriendliness assessment [38, 39]. However, it should be taken into account, that in other regions of the world PBT/vPvB rely on other criteria and regulatory consequences for endocrine disruptors may be different than within Europe and thus those criteria may not be applicable to those regions. In other words, the tool can still be used but the cut‐off criteria will not be applied (in case of ED) outside of the EU region or may be adapted according to country‐specific criteria and thresholds (i.e. in case of PBT/vPvB).

### Ecotoxicological ranking of compositions

Once the overall environmental hazard score of one or more individual components is known, they can be composed for a sunscreen preparation. The mixture can be evaluated for its overall environmental impact. In order to account for the concentration of the respective component, its environmental hazard score is multiplied by its concentration (in wt%) in the sunscreen product.

The results are summed up resulting in a value designated as (ecorank)_i_. The theoretical maximum ranking of the composition *i* is named (max‐ecorank)_i_. Relating (ecorank)_i_ to (max‐ecorank)_i_ yields the relative ecotoxicity for the given composition. In order to turn that relative ecotoxicity into a scale of ecofriendliness, the following transformation is made:

(1) $${\left(\%\mathrm{eco}‐\mathrm{friendliness}\right)}_{i}=\left\{1‐\frac{{\left(\mathrm{ecorank}\right)}_{i}}{{\left(\max‐\mathrm{ecorank}\right)}_{i}}\right\}∙100=a$$

The higher the percentage, the friendlier is the sunscreen formulation for the environment. This calculation method enables a comparison of the environmental impact of different sunscreen formulations and allows for an optimization of the UV filter system with regard to environmental aspects. In order to take the efficiency of a given UV filter composition in terms of achieving a certain SPF value and UVA‐PF value into consideration, the value of the ecofriendliness is multiplied by the filter efficiency, which is the sun protection factor (SPF) and UVA‐protection factor (UVA‐PF) divided by the total filter concentration in wt% (c_UV Filters_). The EcoSun Pass value can be calculated as follows:

(2) $$\text{EcoSun Passvalue}=a∙\frac{\mathrm{SPF}+\mathrm{UVA}‐\mathrm{PF}}{c_{\mathrm{UV}‐\mathrm{Filters}}}$$

Wherein ‘a’ corresponds to the ecotoxicological evaluation of the used filter system obtained from Equation (1). ‘SPF’ and ‘UVA‐PF’ were derived from the sunscreen simulator. Sunscreen simulators calculate the SPF and the UVA‐PF based on a data set with quantitative UV spectra of the relevant UV filters, a mathematical description of the irregularity profile of the sunscreen film on the skin, and the consideration of changes in UV filter concentration due to photostabilities [40].

### Example calculations and threshold criterion

The usefulness of the ESP tool was tested using nine example UV filter compositions resulting in SPFs of 30 (five compositions) and 50 (four compositions), respectively. In order to drive innovation for more ecofriendly sunscreen products, the existing compositions were evaluated according to the EcoSun Pass criteria. Based on this evaluation, the threshold was set to 200. A sunscreen formulation is regarded as ecofriendly if the resulting ESP value is greater than 200. This threshold could be achieved using the best available UV filters currently on the market.

At this time, only few of the available compositions within the European sunscreen market can be considered as ecofriendly. In fact, in 2019 55% of the sunscreens contain an UV filter for (i.e. octocrylene) which the cut‐off criteria was applied [41]. Another 12% of the market share relates to compositions containing a specific UV filter (i.e. zinc oxide) with an unfavourable ecotoxicological profile. Usually, this UV filter is not used in combination with the one where cut‐off criteria were applied.

For the remaining 33% of products on the market, the UV filter compositions were analysed based on the INCI declaration on the product label.

Based on this evaluation it was concluded that about 10% of the sunscreens within the EU market in 2019 showed an ESP value of >200 and can therefore be considered as ecofriendly.

In the following, we will use the term ecofriendliness, which correlates with the quantity given by the ESP value but is a more general expression.

## Results

### Environmental hazard score of co‐formulants

The evaluation of the environmental hazard score of emollients, preservatives and thickeners revealed typical ranges from 1.5–2.25, 2.0–2.75 and 1.5–2.25, respectively (data not shown).

### Environmental hazard score of the UV filters

**Figure 1:**
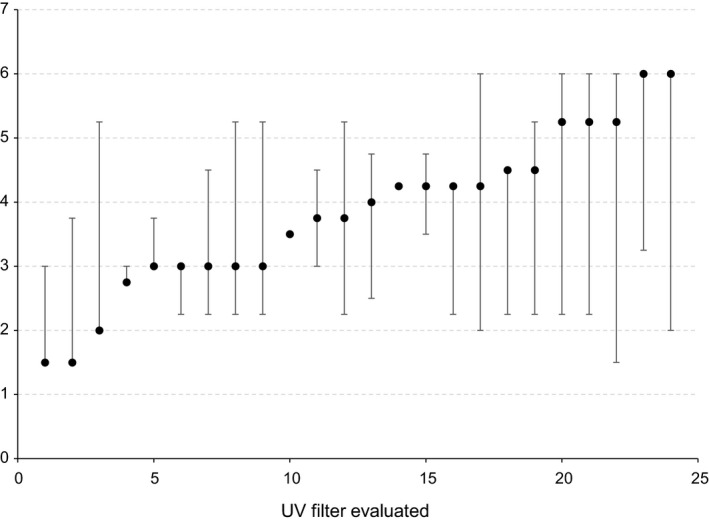
Environmental hazard score of the 24 UV filters evaluated (real (●), best (lower range) and worst case (upper range).

The 24 UV filters evaluated reveal an environmental hazard score (real case) ranging from 1.5 (best/worst case range: 1.5–3) to 6 (best/worst case range: 2–6) allowing for a clear discrimination of the environmental profile of the individual components (Figure 1).

It has to be mentioned, that the numbering of the filters as shown in Table 1 is not identical to those displayed in Figure 1 as the aim of this work was to demonstrate the applicability of this tool rather than providing a detailed ranking of the individual filters.

Furthermore, each individual UV filter has a specific environmental profile indicated by the real case which is build based on available experimental data, valid QSARs and to some extent even based on expert judgement in order to allow for a complete data set evaluation (real case). Since expert judgement and even QSAR may not provide a final conclusion on certain environmental chapters, a best/worst case range was provided in addition to the real case to demonstrate the range of possible results from any new experimental data.

In fact, the best‐ and worst‐case evaluation reveals hazard score ranges, which vary among the UV filters, as expected, as they strongly depend on the individual data availabilities. Thus, the more data are available, the lower is the uncertainty, which then results in a narrow range of variability as shown for substances no. 4 and 15 (Figure 1). On the other hand, substances with a limited data set show a large variability and thus a high uncertainty of the ‘real case’ environmental hazard profile. Examples of the latter category are substances no. 18, 21, 22, 23, and 25, for which additional data are needed in order to narrow down the real situation.

For instance, UV filters with similar efficacies (i.e. ethylhexyl triazone (ETH) and diethylhexyl butamido triazone (DBT)) with <E11> of 420 and 451, respectively), differ in their environmental hazard profile (3 versus 5.25 for the real case).

### Environmental hazard score of other excipients used in sunscreens

As indicated above, the environmental profile of further substances used in sunscreen products such as emollients, thickeners or preservatives is very much consistent among the different functionalities in the products, resulting only in minor variations of the individual environmental hazard scores, not allowing for a clear discrimination among the individual components (data not shown).

### Cut‐off criteria

None of the 24 UV filters investigated meets the criteria for being a PBT and/or vPvB‐substance based on the existing data and conclusions. However, one UV filter meets the criteria for being toxic to the aquatic environment (NOEC < 0.01 mg/L) thus the cut‐off criteria were applied to this substance. For the other 23 filters remaining, a pure hazard‐based ranking was carried out.

### Ecotoxicological ranking of compositions

Based on the chosen examples, the number of different UV filters required to prepare a sunscreen composition with an SPF of 30 ranges from four to five; however, the overall concentrations of UV filters needed for this SPF vary from 11.0%, 12.5%, 17.0%, 25.5%, to 28.0% for example compositions no. 4, 5, 3, 2 and 1, respectively (Table 3). On the other hand, the ESP values range from 0, 82, 143, 216 to 244 for example No. 1, 2, 3, 4 and 5, respectively, showing an increase in ecofriendliness with increasing formulation numbers (i.e. from 1 to 5). Taking into account that ecofriendly sunscreen products obtain an ESP value of >200, two out of the five formulations with an SPF of 30 can be regarded as ecofriendly.

**Table 3:** Ecofriendliness of five representative sunscreen formulations with a sun protection factor (SPF) of 30.

Formulation	Example no. 1		Example no. 2		Example no. 3		Example no. 4		Example no. 5	
Concentration of a specific UV filter	Filter^1^	Conc. [%]	Filter^1^	Conc. [%]	Filter^1^	Conc. [%]	Filter^1^	Conc. [%]	Filter^1^	Conc. [%]
1	EHS	5.0	EHS	5.0	EHS	2.5	EHT	2.5	EHT	2.0
2	HMS	5.0	EHT	2.5	BEMT	2.5	BEMT	2.5	BEMT	2.5
3	OCR	10.0	BEMT	3.0	PBSA	2.0	PBSA	2.0	BEMT (aqueous form)	1.0
4	B‐3	5.0	HMS	10.0	BMDBM	5.0	DHHB	4.0	MBBT	3.0
5	BMDBM	3.0	BMDBM	5.0	‐	‐	‐	‐	DHHB	4.0
Overall UV filter Concentration [%]	28.0%		25.5%		17.0%		11.0%		12.5%	
SPF calculated	30.4		30.9		32.3		30.7		31.5	
UVA‐PF calculated	10.5		10.6		10.1		12.9		24.1	
EcoSun Pass Value	0		82		143		216		244	

^1^ See Table 1 for UV filter names.

To compose a sunscreen with a high SPF (i.e. SPF of 50), four to six UV filters are required. Although the overall UV filter concentration is similar to that of the lower SPF ranging from 13.5% to 27.5%, the higher SPF is achieved by just using more efficient UV filters (Table 4). The resulting ESP value ranges from 0 to 244, with example composition no. 4 (ESP value 244; 13.5% overall UV filter content) being the most ecofriendliest formulation. In contrast, composition example no. 1, reveals to be the least ecofriendliest formulation (ESP of 0, due to the applied cut‐off criterion for aquatic toxicology) containing also UV filters with low efficacy (24% of the 28% total UV filter concentration). Again, the ESP tool allows for a clear differentiation between ecofriendly (50%) and non‐ecofriendly (50%) sunscreen formulations even for high SPFs.

**Table 4:** Ecofriendliness of four representative sunscreen formulations with a sun protection factor (SPF) of 50

Formulation	Example no. 1		Example no. 2		Example no. 3		Example no. 4	
Concentration of a specific UV filter	Filter^1^	Conc. [%]	Filter^1^	Conc. [%]	Filter^1^	Conc. [%]	Filter^1^	Conc. [%]
1	EHS	5.0	EHS	5.0	EHS	5.0	EHT	2.0
2	BEMT	2.0	EHMC	10.0	EHT	2.5	BEMT	2.5
3	TBPT	3.0	EHT	2.5	BEMT	1.0	MBBT	6.0
4	OCR	10.0	MBBT	2.0	MBBT	2.0	TBPT	3.0
5	BMDBM	4.0	DHHB	8.0	DHHB	4.0	‐	‐
6	‐	‐	‐	‐	TBPT	3.0	‐	‐
Overall UV filter Concentration [%]	24		27.5		17.5		13.5	
SPF calculated	53		54		53		57	
UVA‐PF calculated	23.8		18.2		17.7		18.1	
EcoSun Pass Value	0		160		228		272	

^1^ See Table 1 for UV filter names.

The UVA protection factor (UVA‐PF) for the five SPF 30 formulations ranged from 10.1 to 12.9 for formulation No. 1, 2, 3 and 4, respectively, but was about 24.1 for formulation No. 5. At SPF 50, the highest UVA‐PF of 23.8 was achieved with sample formulation No. 1, whereas the other formulations were in the range of 17.7–18.2.

## Discussion and conclusion

The evaluation of the environmental hazard profile of various compounds used in sunscreen products revealed that UV filters are the key components with regard to discrimination between ecofriendly and non‐ecofriendly formulations. The additionally required higher tier data during the ECHA Community Rolling Action Plan (CoRAP) process will provide further valuable information on the environmental impact of UV filters including both sediment and soil organisms and thus allows for an adequate update of the current hazard evaluation status. But already the current evaluation shows differences in the environmental hazard score between UV filters of similar efficacies ranging from rather environmental non‐hazardous (i.e. ethylhexyl triazone, environmental hazard score of 3.0) to environmentally rather hazardous substances (i.e. diethylhexyl butamido triazone, environmental hazard score of 5.25). Likewise, for the inorganic UV filters, a large data set already exists leading to markable differences in the environmental hazard score with 2.75 and 4.25 for TiO_2_ and ZnO, respectively. The results also showed that the amount of available data on the one side and the absence of some so‐called higher Tier data (i.e. chronic aquatic, terrestric and sediment toxicity) does hinder the evaluator from a science‐based evaluation since provisionally conclusions on existing data gaps can be made from existing data (screening level). This already common practice at the European Chemical Agency (ECHA), national authorities and non‐governmental organizations (NGOs) to select for substances on a more profound assessment on already available screening criteria [39, 42]. The additionally provided uncertainty range is not a limitation within the assessment, but rather a supportive and very useful add‐on. It clearly shows whether the current environmental hazard score can be improved by additional (new) data or whether it will remain at least at the existing level.

Nevertheless, it has to be acknowledged, that the additional experimental data may exceed requirements of actual chemical legislations (i.e. in case of Endocrine Disruption, ED), may require legal permissions (i.e. in case of vertebrate studies) or may even go beyond a tonnage based data requirement, which is a current regulatory scheme of many national authorities. In addition, it must be considered, that rather more than less ecotoxicity/environmental/toxicity data will be required as the science of the potential impact evolves.

Although the EcoSun Pass favours valid experimental data on all six environmental chapters, it can also be applied by using the limited experimental data in combination with QSARs and expert judgements.

Ultraviolet filters reveal distinct differences in their efficiency, that is their UV light absorbing potential, which finally impacts the concentration of the individual UV filter in the sunscreen formulation and moreover impacts the achievable SPF (both UVA and UVB) [43]. The combination of both relevant aspects (i.e. environmental hazard score and efficiency) via the EcoSun Pass allows for a clear discrimination of various sunscreen formulations and the filters used with respect to their environmental friendliness. The given example formulations (SPF 30 and 50) show a clear negative correlation between the overall amount of UV filters in the sunscreen product and its ecofriendliness. In fact, formulations with the highest ESP values have the lowest overall UV filter content. This is certainly due to the fact, that the UV filters used in these formulations are higher efficient, but did reveal the lowest environmental hazard score (e.g. no effects in all available tests).

So, the combination of both properties (e.g. hazard and efficacy) fosters the selection for most ecofriendly UV filter candidates. Thus, the ESP tool is applicable to various SPF classes of sunscreen products covering the typical range of SPF being currently present on the market and allows for the selection of most ecofriendly and efficient UV filters.

Taking that into account, the EcoSun Pass tool contributes to other previously published science‐based environmentally evaluation tools such as the ecodesign methodology or the ecoefficiency analysis [22, 44] because it combines hazard and efficiency profiles of UV filters in sunscreen products. While the ecodesign methodology focuses on the aquatic environmental impact profile of cosmetic formulae [22], the EcoSun Pass in addition evaluates the UV filters and all environmentally relevant compartments (i.e. also sediment and soil). Therefore, it goes beyond environmental classification and labelling aspects, which are limited to aquatic toxicity only. Due to the physical–chemical and environmental fate properties of the investigated UV filters, besides water, other compartments such as sediment and soil are deemed to be relevant, and thus the ESP even goes beyond the EU ecolabel for cosmetic rinse‐off products [23]. Any hazard that may occur in corresponding toxicity tests will not contribute to classification and labelling (see also ECHA recommendation on the hazard definition). The EU‐ecolabel approach would lead to a bias in the environmental assessment of the UV filters towards the aquatic compartment, as it neglects hazards in terrestrial and/or sediment‐dwelling organisms. Therefore, the EcoSun Pass tool includes all relevant environmental compartments on an equal proportion, allowing for a more balanced assessment of the UV filters with respect to the environmental hazard score. In addition, specific toxicity may be traced back to the compartment/issue of concern (e.g. bioaccumulation; metabolites; chronic, sediment or soil toxicity), helping to identify the need for further clarification/actions. The applicability of the cut‐off criterion for aquatic toxicity, furthermore, allows for the exclusion of UV filters having negative impact on both freshwater and marine organisms and thus specifically includes the potential effects on corals. In contrast, other tools like the ecoefficiency analysis look for the whole life cycle of a product or chemical, including all steps from the cradle (raw materials) to the grave (waste) and thus are a high‐level environmental profiling [44]. Besides intrinsic toxicological and ecotoxicological information, the ecoefficiency analysis includes general safety (i.e. the number of incidences during production of a substance and/or material) and costs aspects to establishing an ecoefficient portfolio [44]. Under REACH, a socio‐economic SEEbalance approach in addition combines the ecoefficiency analysis with socio‐economic aspects [45, 46]. Hence, this approach requires an in‐depth and detailed analysis of a certain substance or a certain group of substances. As a consequence, it will likely only be carried out on a case‐by‐case decision and for substances subject to authorization under REACH. For the ecological/environmental assessment of a specific sunscreen formula, the requirements of data knowledge are probably too high.

Other environmental evaluation tools like Nordic Ecolabelling or regulation under OSPAR focus on the environmental profile of individual substances used in various consumer or technical applications only and omit the performance criteria of these chemicals [47, 48].

Sunscreen products, however, are complex formulations, relying on a given UV filter composition in combination with a sufficiently high sun protection factor, which is best evaluated using the EcoSun Pass tool. The tool allows for a regular adaption of the individual UV filter hazard profile based on newly available data, and so it can be adjusted easily to reflect the state‐of‐the‐art information. In addition, a ‘real case’ calculation reflects the current environmental hazard profile, whereas additional ‘best’ and ‘worst case’ evaluations indicate the given uncertainty in the current assessment. By saying so, a broad uncertainty range indicates a rather limited amount of hazard information, whereas a narrow range will be given for a well‐investigated UV filter. This information is of particular interest for cosmetic industry in order to select for the most ecofriendly candidates on a short term. Furthermore, it provides a mid‐term perspective on how certain the current environmental hazard evaluation is with respect to potential experimentally based data gaps. Especially, the latter one may cause serious detrimental effects on the environmental profile of a cosmetic formulation if additional data increase the hazardous properties of the UV filter at a later stage. In addition, this tool can be also used outside of the EU region by adapting the selection of approved UV filters in a certain regulatory environment (i.e. United States, Japan) or the cut‐off criteria. The latter one may even be dismissed in some regions (i.e. ED in United States) due to the lack of applicability in that region.

On the other hand, the EcoSun Pass may be also used by manufacturers of UV filters to further evaluate the hazard profile of their filters by identifying and filling data gaps through additional adequate studies.

To conclude, the EcoSun Pass is a suitable tool to select most ecofriendly and efficient UV filters for the ecofriendly sunscreen products. Although the current focus of this tool is on UV filters as the key ingredients of sunscreens, the range of cosmetic products also includes other functional ingredients, which may be evaluated in a similar manner.

## Competing interests

The authors declare that they have no competing interests.
